# PEDOT Coated Thick Film Electrodes for In Situ Detection of Cell Adhesion in Cell Cultures

**DOI:** 10.3390/bios8040105

**Published:** 2018-11-02

**Authors:** Heike Bartsch, Ralf Peipmann, Maren Klett, Dana Brauer, Andreas Schober, Jens Müller

**Affiliations:** 1 Technische Universität Ilmenau, Institute of Micro- and Nanotechnologies, Gustav-Kirchhoff-Straße 7, 98693 Ilmenau, Germany; ralf.peipmann@tu-ilmenau.de (R.P.); maren.klett@tu-ilmenau.de (M.K.); andreas.schober@tu-ilmenau.de (A.S.); jens.mueller@tu-ilmenau.de (J.M.); 2 Technische Universität Ilmenau, Department of Nanobiosystem Technology, Institute of Chemistry and Biotechnology, Prof.-Schmidt-Straße 26, 98693 Ilmenau, Germany

**Keywords:** thick film electrodes, LTCC, PEDOT, impedance, spatial resolution, in situ monitoring, cell adhesion, in vitro cell culture, electroactive cells, neuronal cells

## Abstract

Low temperature cofired ceramics (LTCC) provide a technology for the 3-dimensional integration of sensor arrays into bioreactors covering dimensions of several hundred micrometers. Since optical control in such assemblies is not possible, the in situ detection of cell adhesion on impedance electrodes with high spatial resolution would deliver crucial information. A current limitation is the increasing impedance of microelectrodes with decreasing diameter. This study evaluates the suitability of thick film gold electrodes, pristine and coated with electropolymerized poly(3,4-ethylenedioxythiophene) (PEDOT), for the detection of cell adhesion on the electrode surface. The impedance as criterion for cell attachment is measured with a recording system for electroactive cells with the aim of improving usability. Two cell cultures with different adhesion characteristic are used for adhesion assessment on planar test chips. The impedance increase measured on individual PEDOT coated electrodes due to tight contact of cells reaches a factor of 6.8 in cultures of well-adherent HepG2 cells. Less adhered NG108-15 cells produce a maximum impedance increase by a factor of 2.6. Since the electrode impedance is significantly reduced by PEDOT coating, a reduction of the electrode diameter to values below 100 µm and spatially resolved detection is possible. The results encourage further studies using PEDOT coated thick film electrodes as bio-electronic-interfaces. We presume that such miniaturized electrodes are suitable for 3-dimensional recordings in electroactive cell cultures, providing information of local cell adhesion at the same time.

## 1. Introduction

Planar multi electrode arrays (pMEAs) based on thin film technology are widely used for the investigation of neuronal tissue [1]. However, the established structuring methods are inappropriate for fulfilling the requirements driven by 3-dimensional cell culture technology since assembly in the third dimension is limited. This fact has initiated the development of 3-dimensional multi electrode arrays (3D MEAs) based on low temperature cofired ceramic (LTCC) technology [2]. The approach allows the assembly of thick film electrodes encompassing a volume range of several hundred micrometers. The 3D MEA prototypes monitored primary rat hippocampal and cortical cell cultures over a period of 6 months [2]. In previous works, two limiting usability aspects were unveiled: (i) the impedance of the used thick film gold electrodes (TFGe) increases strongly when the diameter decreases [3] and (ii) optical observation is not possible at perpendicular electrodes.

The first mentioned aspect must be solved because small electrode diameters are needed to ensure adequate spatial resolution of recordings. Functionalization can improve the impedance characteristic [4].

The second aspect causes problems in cell culture monitoring. Neuronal cells require several days until they start bursting [5] and they can remain inactive under disadvantageous conditions. The reason for absent spikes can lie in inactivity or in poor contact of cells with the electrode. Since optical control of cell adhesion at the electrode is impossible, alternative methods are necessary to assess a good electrical contact and improve usability of the system.

A smart solution would be the use of the recording electrodes itself for the detection of cell attachment on their surface. In principle, impedance spectroscopy can tackle this issue. Biomass attached to an electrode entails a change of the local ionic environment and results in a parameter change of the constant phase element, which changes the impedance characteristic of electrodes [6,7,8,9]. The comparison of pristine gold electrodes with poly(3,4-ethylenedioxythiophene) polystyrene sulfonate (PEDOT:PSS) coated ones revealed an impedance decrease, which enabled reliable cell growth detection [10]. However, the cited works have used mesoscale electrodes or interdigital structures with huge area requirement that are not suitable for spatially resolved measurements. The present work aims to investigate the impedance change on thick film microelectrodes with diameters below 250 µm due to cell adhesion in order to assess their miniaturization capability. Since the impedance of a cell layer is in the range of some kΩ [7], a first step towards the application would be a reduction of the initial electrode impedance. Electrodeposition of poly(3,4-ethylenedioxythiophene) (PEDOT) should decrease the impedance of TFGe [11]. The suitability of pristine TFGe and such coated with PEDOT (PEDOTe) for adhesion detection were compared and the electrode size was varied in order to explore the miniaturization limits.

With the purpose of modelling different adhesion behavior independent from cell number, two human cell lines were selected. Hepatocellular carcinoma cell line (Hep-G2) is known to easily form a dense lawn on different surfaces. In contrast, the adhesion behavior of neuroblastoma glioma hybrid cell line (NG108-15) correlates with acidification of the medium during growth and surface properties [12]. Culture conditions that lead to worse adhesion can thus be realized, for example, when the usual laminin coating is omitted [13,14]. In this case, the cells attach by forming globular cell clusters.

Since planar design facilitates the inspection of the electrodes, pMEAs containing either TFGe or PEDOTe were used. The used measurement system foreseen for neuronal recordings allows impedance measurements. The impedance of the microelectrodes with different diameter was captured over the culture time and possible electric parameters for adhesion detection were identified. The spatially assigned impedance increase was evaluated and compared with cell adhesion observed with a scanning electron microscope (SEM).

## 2. Materials and Methods

### 2.1. LTCC Planar MEA

#### 2.1.1. Fabrication

**Figure 1:**
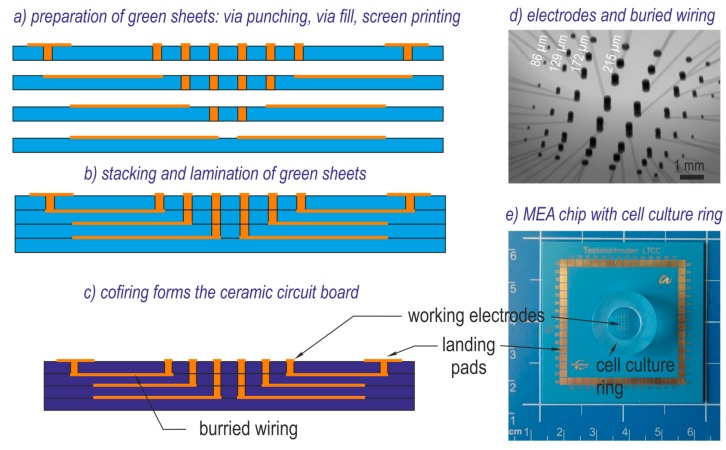
Planar multi electrode array (pMEA) made of low temperature cofired ceramic (LTCC): (**a**–**c**) fabrication steps; (**a**) processing of single green sheets (Green Tape^TM^ 951, via paste 5738, conductor paste 5740A, DuPont); (**b**) stacking and lamination of the unfired foils; (**c**) firing; (**d**) X-ray micrograph of the electrode array showing microelectrodes with different diameter; (**e**) pMEA chip with mounted cell culture ring.

In order to facilitate fabrication and later SEM investigation, pMEAs including thick film electrodes identical with those used for 3D MEAs were used for the feasibility test. LTCC processing uses cost-effective methods for the creation of multilayer devices. Green foils (Green Tape^TM^ 951, DuPont de Nemours, Wilmington, DE, USA) consisting of ceramic and glass powder fractions were embedded in an organic matrix. The technology can use foil-structuring methods, such as punching, blade cut, or laser cut as well. Vias filled with conducting paste connected the single layers and allowed a dense assembly of electrodes. Printed conductor paths realized the electrical wiring. The isostatic lamination of the green sheets applying pressure at temperatures around 70 °C formed a compact green body. During sintering (875 °C, 30 min), the organics burned out and the remaining ceramic and glass components formed a solid ceramic wiring board with excellent electrical isolation. Figure 1a–c illustrates the fabrication process. The ceramic pMEA contained electrodes, buried wiring, and landing pads for the connection of the measurement equipment. The high thickness of the dielectric between signal lines guaranteed minimum capacitive crosstalk. The TFGe varied in size for the purpose of assessing the miniaturization limits. The final electrode diameters (86 µm, 129 µm, 172 µm, and 215 µm) were from the available via punch tools and the shrinkage. Figure 1d depicts an X-ray image of wiring and electrodes. Figure 1e shows the whole chip assembly with mounted cell culture ring, which serves as a fluid reservoir for electropolymerization and cell culture experiments. This ring consisted of fused silica. It was attached with PDMS (Silguard 184, Dow Corning, Midland, MI, USA) on the LTCC surface. Planar MEAs with TFGe are referred to as TFG-MEA in the following.

Potentiostatic electropolymerization of PEDOT creates the functionalization layer on the TFGe using the assembled pMEA as the deposition cell. The reservoir was filled with the electrolyte, containing 0.5 molar aqueous solution of sulfuric acid, 10 mmol/L PEDOT 97% (Sigma Aldrich, St. Louis, MO, USA), and 34 mmol/L sodium dodecyl sulfate (SDS, Sigma Aldrich, St. Louis, MO, USA) as the wetting agent. The voltage amounts to 0.9 V and was applied for 1 min between the working electrodes and a platinum counter electrode. The thickness of the dense layers, calculated on the basis of the logged current density during electropolymerization, was 390 nm ± 134 nm. All chips were cleaned with deionized water after deposition. The MEA chips are hereinafter referred to as PEDOT-MEA.

#### 2.1.2. Electrode Topography

**Figure 2:**
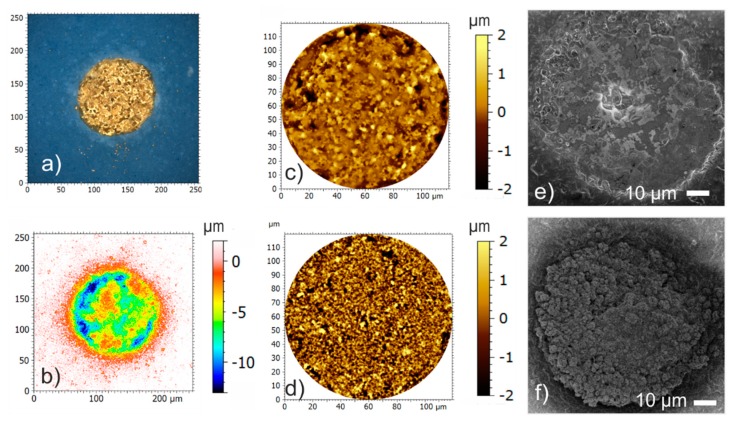
Surface morphology of thick film electrodes: (**a**) laser scanning microscopy (LSM) micrograph of thick film gold electrodes (TFGe); (**b**) contour map of the typical surface topography; (**c**) surface roughness image of TFGe (Sq = 0.484) and (**d**) of TFGe coated with PEDOT (PEDOTe) (Sq = 0.939); (**e**) SEM micrograph of the surface structure of TFGe and (**f**) of PEDOTe. PEDOT = poly(3,4-ethylenedioxythiophene).

The surface structure of the electrodes was studied with laser scanning microscopy (LSM, Olympus OLS4100, Olympus Europa SE & Co. KG, Hamburg, Germany) and electron scanning microscopy (SEM, S 4800-II, Hitachi High-Technologies Europe GmbH, Krefeld, Germany). The captured LSM data were processed with the software MountainsMap^®^ (Digital Surf, Besançon, France). The real color image of a TFGe is depicted in Figure 2a. A grainy surface structure is visible. For height profile evaluation, invalid measuring points (sparks) were approximated with an adapted curve. Figure 2b depicts the resulting topography graph of a typical TFGe. For roughness calculation, a Gaussian filter (DIN EN ISO 16610-71) with a cut-off length of 8 µm was applied. The resulting area profiles of the roughness are shown in Figure 2c,d. Figure 2e,f present SEM micrographs of a TFGe and a PEDOTe.

The irregular topography of the TFGe (Figure 2a,b) is a consequence of the paste-based process. PEDOTe have similar topography, since the layer thickness is in the order of magnitude of the roughness of the underlying TFGe (Figure 2c). The increased roughness of the PEDOTe (Figure 2d) is caused by the formation of fractal structures, visible on the SEM image in Figure 2f. Indeed, the SEM image of TFGe in Figure 2e reveals the grainy structure of the fired paste, but no fractal structure. It is thus assumed that porosity increases the effective thickness, in particular at the electrode circumference as visible in Figure 2f, entailing an increase of the effective electrode area.

### 2.2. Impedance Measurement

**Figure 3:**
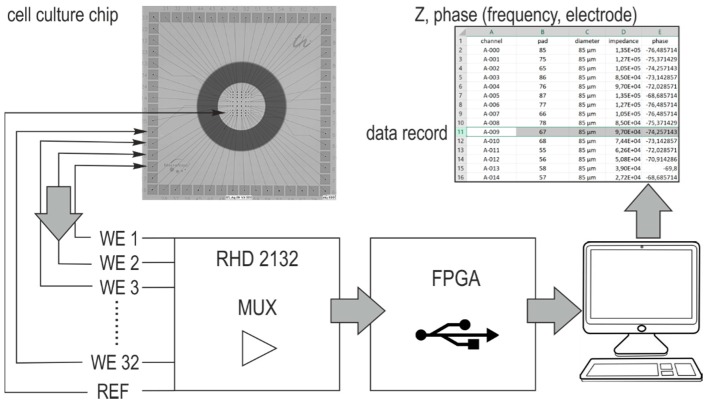
Schematic of the impedance measurement setup. RHD 2132: chip for recording of biopotential signals, WE = working electrode, REF = reference electrode, MUX = multiplexer, FPGA = field-programmable gate array board, Z = impedance.

The buried screen-printed conductor lines connect the working electrodes over landing pads with pin contacts on the adapter board carrying the acquisition chip (RHD 2132 from Intan Technologies, Los Angeles, CA, USA). The measurement adapter is described in previous works [2]. It serves for recordings in electroactive cell cultures and provides impedance analysis covering a frequency range between 20 Hz and 5 kHz. The schematic set up in Figure 3 illustrates the information flow. A platinum wire is connected with the reference (REF) of the chip and plunged into the electrolyte. All electrodes are addressed by a multiplexer at a given frequency, and the captured impedance data are transferred to a computer. The single data record contains the position information, frequency, phase, and impedance magnitude. In this way, each electrode can be individually evaluated.

The measurements were carried out in a frequency range between 50 Hz and 5 kHz. Three values were captured at each acquisition point. The software MiniTab17 was used for statistical analysis. It allows classification into defined data groups: data for each day and the respective MEA were electively classified according to frequency or electrode diameter. In this way, a statistical data base including the data of 16 electrodes for each electrode size per MEA was collected. The frequency of 1 kHz served as reference frequency for comparative measurements of different sample treatments.

### 2.3. Cell Adhesion Experiments

#### 2.3.1. Chemicals and Reagents

Sterile phosphate buffered saline (PBS), Minimum Essential Medium (MEM), Dulbecco’s Modified Eagle’s medium (DMEM), Penicillin/Streptomycin, sodium pyruvate, glutaraldehyde, and tergazyme were purchased from Sigma-Aldrich (Steinheim, Germany). Fetal bovine serum (FBS) and glutamine were purchased from Biochrom (Berlin, Germany).

#### 2.3.2. Investigation of Electrode Conditioning Influence

**Figure 4:**
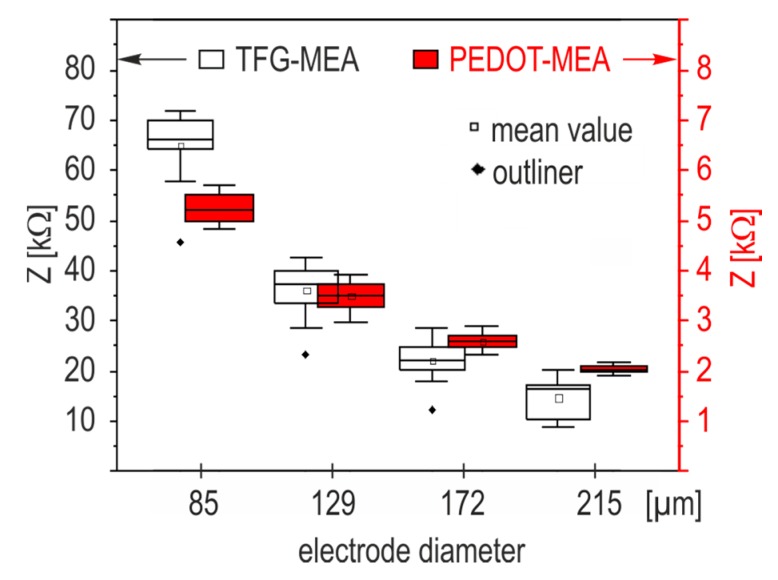
Box plot of impedance magnitude (1 kHz) as a function of the electrode diameter. Planar MEAs with TFGe are referred to as TFG-MEAs. MEA chips with PEDOT are referred to as PEDOT-MEA.

**Figure 5:**
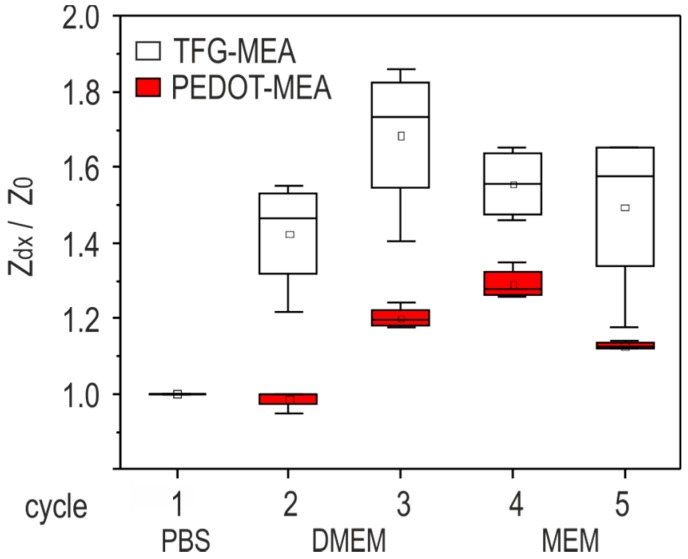
Box plot of impedance magnitude (1 kHz) after cleaning and conditioning steps.

TFG-MEAs and PEDOT-MEAs (two of each electrode type) were cleaned with tergazyme, rinsed in deionized water, and sterilized in ethanol for 1 h under a laminar flow hood. After drying, the chip reservoir was filled with PBS and stored at room temperature for 4 h. After this initial treatment, the first impedance measurement was carried out at the reference frequency of 1 kHz (cycle 1). Figure 4 depicts the boxplots of this initial impedance measurement for both electrode materials at 1 kHz grouped according to electrode diameter. Subsequently, PBS was exchanged by DMEM and the impedance measurement was repeated (cycle 2). After storing for two days in an incubator at 37 °C, the impedance measurement was repeated again (cycle 3). The used MEA chips were cleaned applying the initial procedure, filled with MEM, and the impedance was measured after 4 h dwell time (cycle 4). The last impedance measurement was carried out after incubation of the MEA for two days at 37 °C (cycle 5). The box plots of the impedance values standardized to the initial one obtained in cycle 1 are depicted in Figure 5.

#### 2.3.3. Cell Culture

Brain-derived mouse/rat tumor hybrid cell line NG108-15 (ATCC^®^ HB-12317™) was cultivated in DMEM containing 10% fetal bovine serum (FBS), 2 mM glutamine, 100 U/mL penicillin, and 100 µg/mL streptomycin at 37 °C in a cell incubator at 95% relative humidity and 5% CO_2_. The cell line was passaged every two days.

The human hepatocellular carcinoma cell line HepG2 (ATCC^®^ HB-8065™) was grown in MEM containing 10% fetal bovine serum (FBS), 2 mM glutamine, 100 U/mL penicillin, 100 µg/mL streptomycin, and 1 mM sodium pyruvate at 37 °C in a cell incubator at 95% relative humidity and 5% CO_2_. The cell line was also passaged every two days.

#### 2.3.4. Cell Seeding and Growth on MEAs

Two pMEAs of each electrode material were used for each cell cultivation experiment. Before cell seeding, MEAs were cleaned with tergazyme, rinsed in deionized water, and sterilized in ethanol for 1 h under a laminar flow hood. Before starting the experiment, MEAs were stored in PBS for 4 h. Afterwards, initial impedance was measured with PBS as the electrolyte (Day 0). On day 1, PBS was removed, MEAs were rinsed with distilled water, and cultivation medium was added to the cell cultivation chamber of the MEA. This step served for conditioning of the electrodes in the cultivation medium. The impedance in the presence of cultivation medium was recorded. Then, 35,000 cells (HepG2, NG108-15) were directly seeded on the MEA surface on day 3 and impedance (Z_3_) was measured. In the following days (d4–d7), the impedance of MEAs covered with the growing cell layer was measured daily. Every day the medium on the MEAs was renewed.

After the last impedance measurement on day 7 (Z_7_), viability of cells grown on separate MEAs was analyzed. For that, an Innovatis CASY^®^ Cell Counter + Analyzer system was used. After trypsination, 50 µL of cell suspension was mixed with 10 mL of Casyton solution and the number of total and living cells per mL was determined by measuring cell membrane integrity.

#### 2.3.5. SEM Imaging

Because of the opaque character of the ceramic it was not possible to examine the electrodes with a common transmitted light microscope. Scanning electron microscopy (SEM) provides a suitable method for the investigation of cell adhesion on surfaces. The MEAs were washed thrice with PBS after cell number counting. Only tightly adherent cells remain thus on the surface. These cells were fixed using 2.5% glutaraldehyde at 4 °C for 1 h and 2 times washed with distilled water. Samples were dried, sputtered with a thin platinum layer and examined by scanning electron microscopy (SEM Hitachi S 4800-II, Hitachi High-Technologies Europe GmbH, Krefeld, Germany).

## 3. Results

### 3.1. Influence of Electrode Material and Conditioning on Impedance

Impedance characteristics of electrodes depend on the interface material. Gold is the most used electrode material in BioMEMS and has a Faradaic character, while the conducting polymer PEDOT is characterized by electronic and ionic conducting mechanisms [15]. Impedance measurements represent the resulting variation of the charge transfer through the electrolyte-solid interface. Values measured at 1 kHz in PBS after 4 h storage (Section 2.3.2) were classified according to electrode size. The resulting box plots on the base of all measured electrodes in Figure 4 show that PEDOT coating decreases the electrode impedance by two orders of magnitude and weakens scaling effects significantly. The resulting noise reduction is a strong argument for the use of PEDOT functionalization as an interface material in biologic systems. Electropolymerization with PEDOT increases the miniaturization capability of thick film microelectrodes.

Electrode conditioning allows the electrochemical processes at the electrode surface to reach the equilibrium state. While the impedance characteristic of gold electrodes is dominated by the double layer capacitance [16], ion diffusion is one significant influence on the impedance change of PEDOT coatings [17]. The evaluation of conditioning influences on the electrode impedance provides crucial data for the assessment of the measurement accuracy. Figure 5 depicts impedance data measured at 1 kHz before and after exposure to cell culture media. The values of all measured electrodes in the respective category are compared. Treatment cycles are described in Section 2.3.2. The impedance ratio Z_dx_/Z_0_ is standardized to the value measured in cycle 1 (PBS after 4 h storage).

The results reveal that TFGe are subject to strong variations after medium exchange and during storage in cell culture medium for the first few days. The impedance change during conditioning can reach a ratio of 1.8 in comparison with the initial measurement in PBS. PEDOTe are more robust against such influences: the maximum ratio is below 1.4. The stronger sensitivity of TFGe to medium exposure over time can be attributed to pH changes or degradation of the cell culture medium during incubation. The highly polarizable double layer is prone to such changes. A further influence is the formation of monolayers on the gold surface [16].

### 3.2. Influence of Cultivation Time on Impedance Characteristics

**Figure 6:**
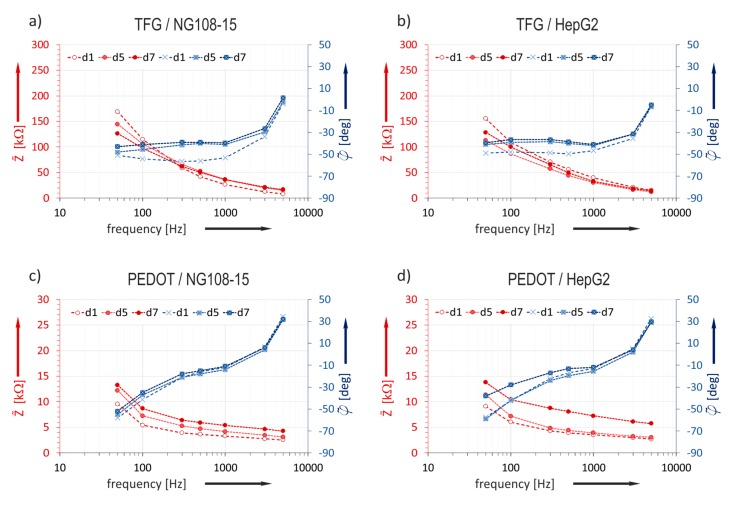
Bode diagrams of median values obtained from measurements in cell culture NG108-15 and HepG2. Frequency varies from 50 Hz to 5 kHz: (**a**) NG 108-15 cultured in TFG-MEAs; (**b**) HepG2 cultured in TFG-MEAs; (**c**) NG 108-15 cultured in PEDOT-MEAs and (**d**) HepG2 cultured in PEDOT-MEAs.

The impedance characteristic was studied over the cultivation time on the base of the daily measured data (Section 2.3.4). The median values $\tilde{Z}$ of the impedance magnitude and $\tilde{\varphi }$ of the phase for data sets measured on TFGe and PEDOTe are presented as bode plots in Figure 6. Data bases are the values of electrodes with 129 µm diameter.

A correlation between the impedance magnitude characteristic of TFGe and cultivation time is not evident. The phase course changes between day 1 and day 5.

On the contrary, the characteristic of PEDOTe reveals a clear tendency: the characteristic lines move evidently towards a higher impedance magnitude as cultivation time passes. An obvious trend occurs at frequencies above 100 Hz for both cell cultures. The phase course is not significantly affected. Since a closer examination of the results should give clearer insight, the ratio between initial impedance directly after cell seeding on the MEAs and the value after a defined cultivation time was studied.

### 3.3. Impedance Increase Dependent on Culture Time

**Figure 7:**
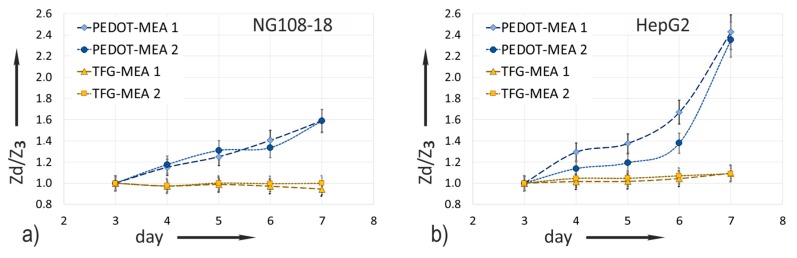
Impedance increase ratio calculated using Equation (1) for TFG-MEAs and PEDOT-MEAs (based on mean values of all electrodes measured at 1 kHz): (**a**) NG108-15 cell culture; (**b**) HepG2 cell culture.

Relating the measured impedance increase to the values captured on the day of cell seeding (day 3) eliminates conditioning influences. The median values $\tilde{Z}$ of the impedance magnitude measured at 1 kHz as a function on the cultivation time (Section 2.3.4.) are the bases of the diagrams in Figure 7. The measurement values of all electrode sizes are considered. The standardized relation *Z_d_*/*Z_3_* uses the respective median $\tilde{Z}$_(d=*x*)_ with *x* = day 3–7 and the reference value $\tilde{Z}$_3_ measured at day 3 directly before cell seeding. Equation (1) gives the relation:

(1) $$\frac{Z_{d}}{Z_{3}}=\frac{{\tilde{Z}}_{\left(d=x\right)}}{{\tilde{Z}}_{\left(d=3\right)}}$$

Referring to the impedance value $\tilde{Z}$_3_ directly before cell seeding at day 3 compensates for conditioning effects. Figure 7a compares the ratio obtained from impedance experiments with NG108-15 cells and Figure 7b those with HepG2 cells.

The impedance ratio measured on gold does not change over the whole cultivation time in both experiments. This confirms the previous made statement based on the bode plots in Figure 6a, that the impedance change of TFGe with small dimensions is not suitable for in situ monitoring of cell attachment.

PEDOT coating makes the increase in impedance due to cell attachment measurable. A large effect is evident. The less adherent cell culture NG108-15 shows a moderate impedance rise. On day 7, the ratio increases by a factor of 1.6. In comparison, the impedance of HepG2 cells increases strongly, in particular after day 6. The maximum ratio of 2.4 emerges on day 7.

At the end of the experiment, both cell lines still showed good viability on both MEA types. There was no significant influence of the electrode material on viability observed. The values varied between 76% and 82% after 4 days of cultivation. The total cell number of NG 108-15 cells was 305,350 ± 25% (TFG-MEAs) and 263,400 ± 2% (PEDOT-MEAs). HepG2 cells total number was 148,200 ± 3% (TFG-MEAs) and 151,300 ± 3% (PEDOT-MEAs). It must be noted here that the cell number does not correlate with the impedance increase. Although the number of NG108-15 cells on day 7 is evidently higher than that of HepG2, the impedance increase is significantly lower. This observation will be explained in the conclusions.

### 3.4. Impedance Increase on Individual Electrodes

**Figure 8:**
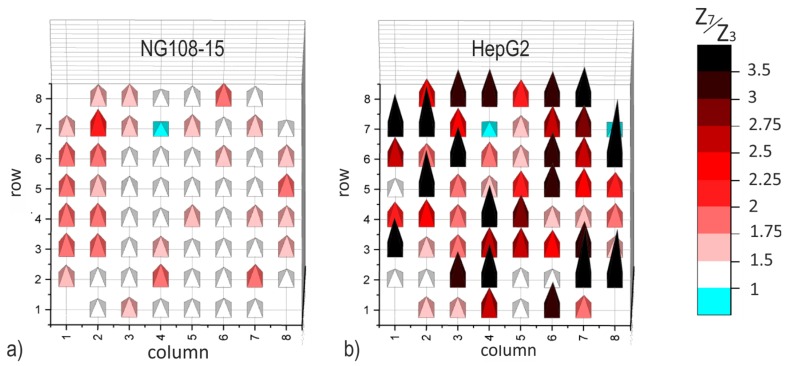
Spatial mapping of the impedance ratio Z7/Z3 (PEDOT-MEAs) measured on single electrodes at 1 kHz: (**a**) NG108-15 cell culture and (**b**) HepG2 cell culture.

The evident dependence of the impedance magnitude on culture time in a comfortable frequency range could be a valuable instrument for in situ monitoring of cell attachment on electrodes. Since TFGe are not sensitive enough, only data captured on PEDOTe were evaluated concerning their individual characteristics. The impedance data of two PEDOT-MEAs are evaluated for each cell culture. The individual electrode impedance on day 7 (Z7) is set in relation to that of day 3 (Z_3_), similar to Equation (1). Figure 8 depicts bar charts of this local impedance ratio Z7/Z3 dependent on the position of the single electrodes. Since the characteristics do not differ significantly, one example is presented for each cell culture in Figure 8. The columns in the graph are assigned to electrodes with equal diameter, rows include electrodes with different diameter (compare Figure 1d). The impedance ratio observed on individual electrodes in cell culture NG108-15 is lower than 2.75. That of cell culture HepG2 is evidently higher and can reach values above 3.5.

**Figure 9:**
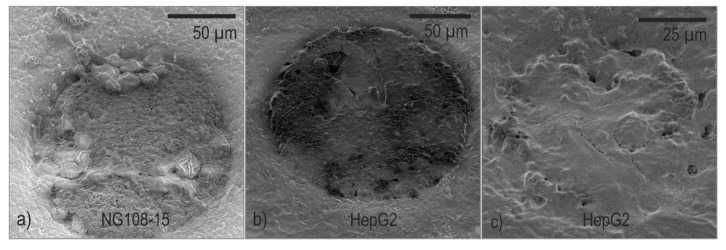
SEM micrographs of fixed cells on PEDOT-MEAs: (**a**) NG108-15 spheroid cell agglomerates; (**b**) Hep-G2 cells partially covering the electrode; (**c**) Hep-G2 cells densely covering a small electrode with 86 µm diameter.

Overview SEM images are provided in Figure S1 (NG108-15) and Figure S2 (HepG2) as Supplementary Material. The observed cell spreading matches well with the impedance maps in Figure 8. Figure 9 shows selected SEM micrographs. Figure 9a depicts an example of an electrode with agglomerates of NG108-15 cells. The image is representative for a good electrode coverage in this study. In general, cells adhesion on the surface is poor and only a few electrodes are covered with more than single cell clusters. The low to moderate local impedance increase correlates well with the impressions gained from SEM micrographs. The maximum value of the impedance ration on single electrodes amounts to 2.65 and was observed on a 129-µm-electrode.

The cell lawn of the HepG2 culture spread well over the surface; only a few electrodes were left uncovered. Some electrodes were partially overgrown as demonstrated in the example in Figure 9b and some are fully covered with a dense cell lawn as shown in the example in Figure 9c. The observed overgrowth correlates with the local impedance ratio. The maximum value of the impedance ratio was 6.8 and occurred on the smallest electrodes with a diameter of 86 µm.

## 4. Discussion and Conclusions

The impedance characteristics of TFGe and PEDOTe are compared in the first part of this work. The results in Figure 4 show that PEDOT coating entails a significant reduction of the impedance magnitude. The impedance magnitude reached values in the range of some kΩ. In addition to the conducting mechanism, the rough surface structure (see Figure 2) is assumed to increase the effective electrode area and contributes to this decrease. Measurements after storage in cell culture media revealed that PEDOTe were less prone to conditioning influences than TFGe (Figure 5).

Since the impedance change caused by cell overgrowth was in the kΩ-range [7], it is plausible that cell tissue related impedance changes on TFG-MEAs in Figure 6a,b were not detectable because of the high initial impedance of TFGe, which was around 20–70 kΩ at 1 kHz, depending on size (see Figure 4). The impedances of PEDOTe amounted to 2–5 kΩ and were in the order of magnitude of the cell related changes. Curves measured with PEDOT-MEAs (Figure 6c,d) reveal a clear tendency. Therefore, only the results based on PEDOT-MEAs were considered in Section 3.4.

**Figure 10:**
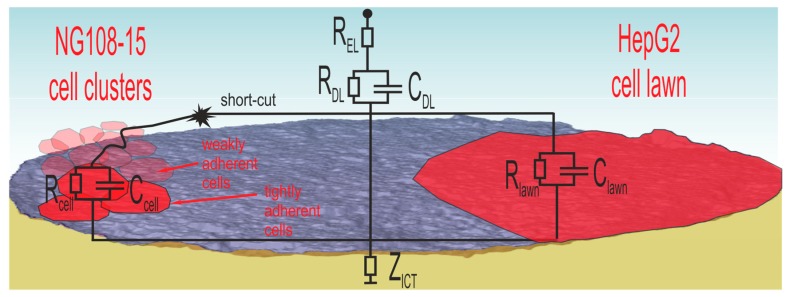
Signal path associated to different cell adhesion scenarios of NG108-15 cells clusters and strong adherent HepG2 cell lawn.

In accordance with the literature [10], the ratio Zd/Z3 at 1 kHZ was a measure for the decreasing portion of the electrode area in direct contact with the electrolyte when cells cover the surface. Figure 7 reveals different characteristics dependent on cell type. From the electrical point of view, only cells with tight contact to the electrode contribute to an impedance increase. Figure 10 illustrates the situation for both cell types.

NG108-15 cells form globular cell clusters. Some clusters adhere tightly at the electrode while others attach weakly on top. These cells contribute to the cell number, but the weak contact enables short-cuts through the surrounding electrolyte. Consequently, the cell number does not correlate with the impedance measurement. The SEM investigation confirms the impedance measurement because the PBS washing steps during preparation removes weakly adherent cells and only the tightly adherent ones, which contribute to impedance changes, remain at the surface.

In the case of the well-spreading HepG2 culture, the cell lawn covers wide areas and electrodes, many of them completely. The dense lawn adheres strongly and is not washed away during SEM preparation. The observed impedance increase in Figure 8b is related to the covered electrode area.

Since the cell number in this experiment is lower than that made with NG108-15, we attribute the impedance increase to the cell adhesion at the electrode. The impression based on SEM pictures (Figures S1 and S2) matches well with the spatial distribution of the impedance ratio in Figure 8.

These promising results must be underpinned by pursuing investigations. Aspects of long-term stability must be tested. Functionality improvement, e.g., by the use of composites of carbon nanotubes and PEDOT (PEDOT-CNT) is a further task. Electropolymerized PEDOT, here tested on pMEAS, can be applied on 3D MEAs in future designs.

The current test shows that the impedance increase caused by cell adhesion is large enough to be detected by PEDOTe, which can be used for signal recordings at the same time. Since the impedance of the thick film microelectrodes with a diameter of 86 µm still did not reach the operation limits of the electronic measurement system, their dimensions can be further decreased. The measurement equipment used for recordings is able to detect the changes, which reached a factor of 6.8 in this experiment. The crucial usability benefit consists in the possibility of testing the tight cell contact with the electronic system prior to the expected start of bursting, saving experimental time and money in the case of failure.

## Supplementary Materials

The following are available online at http://www.mdpi.com/2079-6374/8/4/105/s1.
