# Supplementary material for: PEDOT Coated Thick Film Electrodes for In Situ Detection of Cell Adhesion in Cell Cultures
Source: Biosensors (Basel). 2018 Nov 2;8(4):105. doi: 10.3390/bios8040105 (PMC6315667; doi:10.3390/bios8040105)
Supplement: Supplementary file 1 [file biosensors-08-00105-s001.pdf]

Supplementary Materials

Article

PEDOT Coated Thick Film Electrodes for In Situ Detection of Cell Adhesion in Cell Cultures

Heike Bartsch [1,*], Ralf Peipmann [1], Maren Klett [1,2], Dana Brauer [1,2,*], Andreas Schober [1,2] and Jens Müller [1]

[1] Technische Universität Ilmenau, Institute of Micro- and Nanotechnologies, Gustav-Kirchhoff-Straße 7, 98693 Ilmenau, Germany; ralf.peipmann@tu-ilmenau.de (R.P.); maren.klett@tu-ilmenau.de (M.K.); andreas.schober@tu-ilmenau.de (A.S.); jens.mueller@tu-ilmenau.de (J.M.)

[2] Technische Universität Ilmenau, Department of Nanobiosystem Technology, Institute of Chemistry and Biotechnology, Prof.-Schmidt-Straße 26, 98693 Ilmenau, Germany

* Correspondence: heike.bartsch@tu-ilmenau.de (H.B.); dana.brauer@tu-ilmenau.de (D.B); Tel.: +49-3677-69-3452 (H.B.)

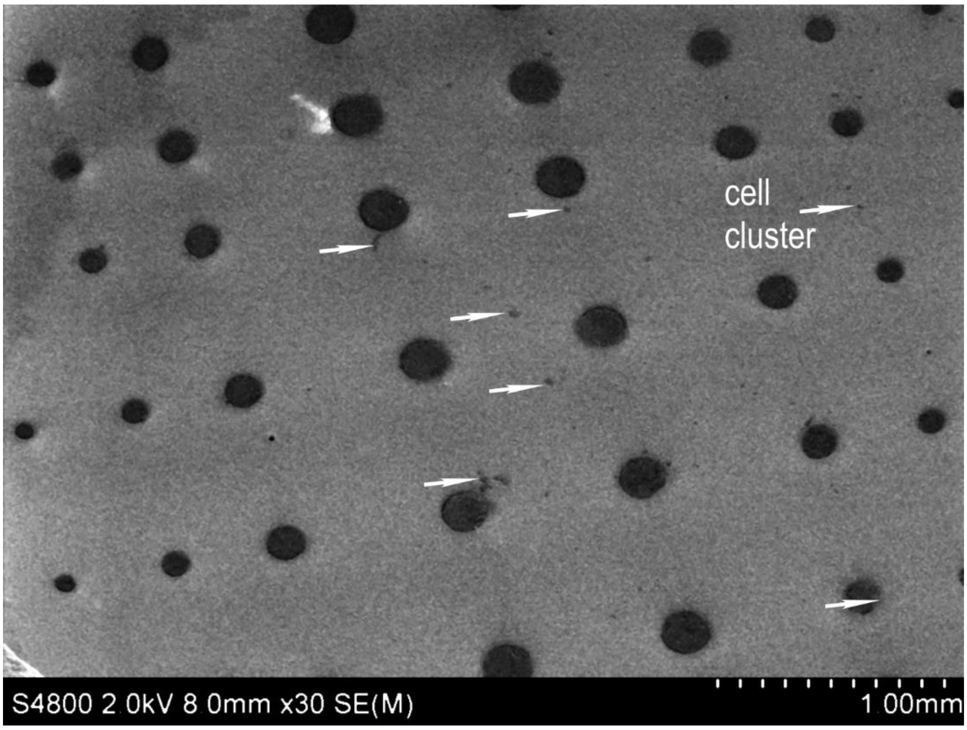

Figure S1. Scattered cell clusters of NG108-15 cells on the surface of a LTCC-MEA with PEDOT coated electrodes.

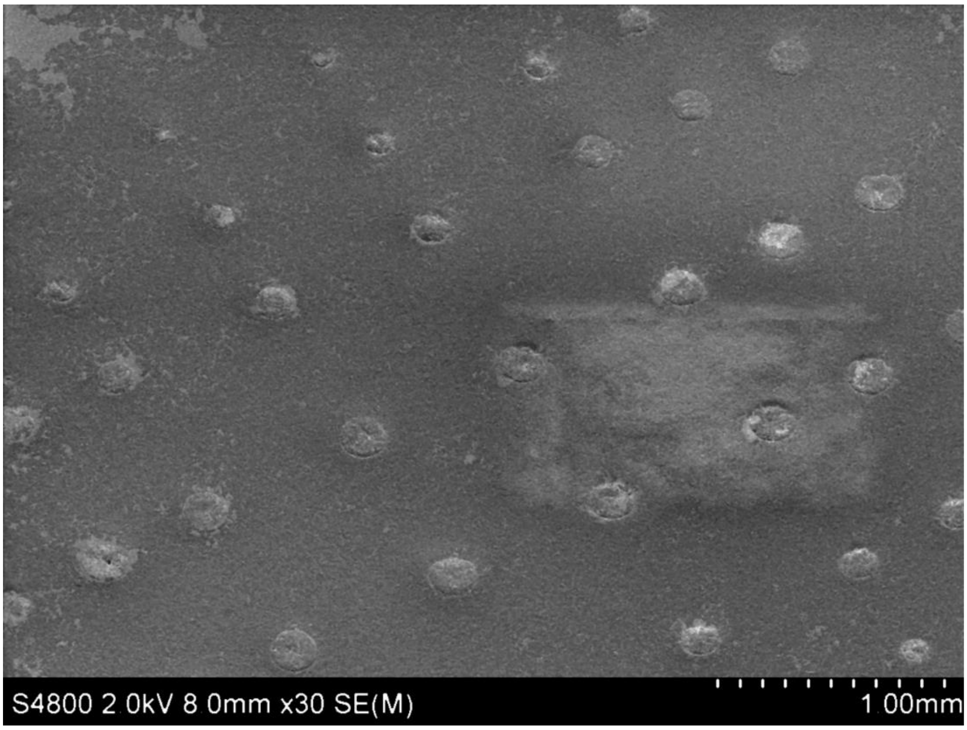

Figure S2. Cell lawn of Hep-G2 cells on the surface of a LTCC-MEA with PEDOT coated electrodes. The bright region results from charging effects due to the insulating substrate.
